# Emerging integrated care models for children and youth with mental health difficulties in Norway: a horizon scanning study

**DOI:** 10.1186/s12913-023-09858-x

**Published:** 2023-08-14

**Authors:** Ida Charlotte Holmen, Sina Waibel, Oddvar Kaarboe

**Affiliations:** 1 University of Oslo, Oslo, Norway; 2 Faculty of Medicine, University of British Columbia, 317 – 2194 Health Sciences Mall, Vancouver, BC V6T 1Z3 Canada; 3 Department of Economics and IGS, University of Bergen, Bergen, Norway

**Keywords:** Mental health services, Child, Adolescent, Integrated delivery system, Continuity of patient care

## Abstract

**Background:**

The implementation of Integrated Care Models (ICMs) represents a strategy for addressing the increasing issues of system fragmentation and improving service customization according to user needs. Available ICMs have been developed for adult populations, and less is known about ICMs specifically designed for children and youth. The study objective was to summarize and assess emerging ICMs for mental health services targeting children and youth in Norway.

**Methods:**

A horizon scanning study was conducted in the field of child and youth mental health. The study encompassed two key components: (i) the identification of ICMs through a review of both scientific and grey literature, as well as input from key informants, and (ii) the evaluation of selected ICMs using semi-structured interviews with key informants. The aim of the interviews was to identify factors that either promote or hinder the successful implementation or scale up of these ICMs.

**Results:**

Fourteen ICMs were chosen for analysis. These models encompassed a range of treatment philosophies, spanning from self-care and community care to specialized care. Several models placed emphasis on the referral process, prioritizing low-threshold access, and incorporating other sectors such as housing and child welfare. Four of the selected models included family or parents in their target group and five models extended their services to children and youth beyond the legal age of majority. Nine experts in the field willingly participated in the interview phase of the study. Identified challenges and facilitating factors associated with implementation or scale up of ICMs were related to the Norwegian healthcare system, mental health care delivery, as well as child and youth specific factors.

**Conclusion:**

Care delivery targeting children and youth’s mental health requires further adaptation to accommodate the intricate nature of their lives. ICMs have been identified as a means to address this complexity by offering accessible services and adopting a holistic approach. This study highlights a selection of promising ICMs that appear capable of meeting some of the specific needs of children and youth. However, it is recommended to subject these models to further assessment and refinement to ensure their effectiveness and the fulfilment of their intended outcomes.

**Supplementary Information:**

The online version contains supplementary material available at 10.1186/s12913-023-09858-x.

## Background

Mental health—or social and emotional well-being—is fundamental to human development and essential for all children to flourish. Investing in the early years of life can improve health and well-being both in midlife and in later years [1]. However, limited access to care, mal-distribution of providers, and lack of coherent policies impede the adequate delivery of mental health care to children and youth [2]. System fragmentation in combination with insufficient communication and linking of services can lead to duplication and gaps in care [3], additional costs to the system and the provision of variable quality of care to children and youth [4]. Integrated care models (ICMs) models where care services are being organised, financed and managed in an integrated and holistic way could counteract the consequences of system fragmentation [4–6].

The core of ICMs is “the provision of the right care, at the right time by the appropriate service provider in a timely manner, irrespective of organizational boundaries or financial flows” [7]. Available ICMs tend to focus on adults [8] and there is a great need to learn more about ICMs specifically for children and youth with mental health difficulties [9]. Children and youth are not “small” adults. They evolve throughout various developmental stages and their dependence on family and social care is different compared to adulthood [10]. As a child grows up, his or her needs for healthcare services differ [10]. Comprehensive ICMs for children and youth include creating holistic support and efficient coordination across different interfaces and disciplines including psychiatrists, psychologists, paediatricians, and social workers in primary and specialty care as well as throughout the developmental stages of a child or youth [6, 10]. Furthermore, it can enhance early intervention and distribution of access and expertise in primary and community care and improve allocation of resources in a more sustainable way [10]. Overall, an integrated care approach to mental health benefits children and youth by providing comprehensive, timely, and coordinated support, improving access to services, and promoting continuity of care.

System fragmentation cannot be solved with increased resources only, and even the richest countries are facing challenges. One example is Norway, a European country that is spending more on health per capita than any other country in the European Union [11]. Nevertheless, the number of children and youth diagnosed with a mental illness is high and increasing [12–14]. For example, the share of girls aged 15 to 17 diagnosed with mental or behavioural disorders – depression, anxiety disorders, adjustment disorders and eating disorder being the most common – increased from 5 to 7% from 2011 to 2016 [12]. There has also been an increase among girls aged 13 to 16 reporting mental health difficulties (16 to 25% from 2010 to 2021), including those that have not necessarily been diagnosed with a mental health illness [15].

On the aggregate level, the Norwegian healthcare system provides universal health coverage, is tax-funded and semi-decentralised, and has a strong primary care focus [10, 11, 16]. The central government is responsible for specialist care, whereas municipalities provide primary and community care [11]. There is a gatekeeping system in place as a patient needs a referral from a general practitioner to access specialist care. Public health services for pre-school and school children are provided in municipal health centres and health centres located in schools [17].

Division of administerial levels and semi-decentralisation creates challenges in obtaining continuity of care and consistent information flow between sectors [11]. In the national escalation plan for children and youth with mental health issues, municipalities highlight a need to broaden out and further develop care services, improve user involvement and increase awareness and knowledge about mental health for children, youth and their families [9]. In the same plan, the central government underlines the importance of implementing various ICMs and the need for transparency in the allocation of responsibilities for mental health service delivery across different levels of care [9].

With this background, the study objective is to summarize and assess emerging ICMs for mental health services targeting children and youth in Norway.

## Research methods

This study employed a horizon scanning methodology, which can be defined as a systematic examination of information sources to detect early signs of important developments [18]. Horizon scanning can be conducted using a wide variety of information sources, such as surveys, media, scientific or grey literature, or individual or expert group opinions [18]. In order to detect early signs and assess potential ICMs, two steps were taken. In step one, ICMs were identified by (a) a review of scientific and grey literature on emerging ICMs for child and youth mental health, and (b) suggestions from experts to find additional ICMs that may have been missed during the review. The identified ICMs were filtered by the research team to meet the general inclusion criteria. In step two, the selected ICMs were assessed by the experts through semi-structured interviews to identify facilitators and barriers to successful implementation or scale up. The project and data collection technique were approved by the Norwegian Centre for Research Data.

### Identification of mental health ICMs for children and youth

In step one, a review of scientific and grey literature was undertaken to search for emerging ICMs for child and youth mental health. Scientific literature was identified by searching PubMed, Google Scholar and databases of the University of Oslo, Norway. Grey literature was located through relevant seminars arranged by the University of Oslo. Norwegian governmental websites such as of the Norwegian Directorate of Health, the Norwegian Directorate for Children, Youth and Family Affairs and the Norwegian Institute of Public Health, as well as municipal websites were searched.

Search words included but were not limited to integrated care for children and youth, youth mental health services, new models of integrated care or mental health innovations. First, titles and abstracts of scientific and grey literature were scanned for relevance to the research topic and then full-text articles were retrieved to revise if inclusion criteria were met. Bibliographies were hand searched for further relevant references. Included were articles or reports that met the following inclusion criteria: (i) they presented an ICM for child and youth mental health services across and within health and social care interfaces in Norway, (ii) they were under development or piloted within the last 10 years, and (iii) they were published in English or Norwegian. The choice of a 10-year limit was based on experiences from other horizon scanning studies aiming to grasp early signs of developments [18, 19].

In step two, key informants were contacted to identify additional ICMs that may have been missed during the literature review. The sampling process consisted of two stages. In the first stage, the study contexts were chosen. Municipalities within the geographical area of South-Eastern Norway and identified in the literature search for having innovative approaches of ICMs were selected. Norway is a long and mountainous country and face challenges regarding equal access to health care across the country, particularly in rural and sparsely populated areas [17]. The choice of study setting is based on both practical reasons and representativeness as South-Eastern Norway covers half of the Norwegian population and encompasses both urban and rural areas (including the capital of Norway, Oslo). In the second stage, key informants were chosen. Firstly, different service centres in selected urban and rural municipalities, aiming to cover all five counties in South-Eastern Norway, were contacted to identify key informants. Then, through a snowballing technique [20], specialist sectors and other organizations got introduced continuously, including child and adolescent psychiatric outpatient clinics and user representative organisations. We relied on maximum-variation sampling [20], i.e., a heterogeneous sample of key informants representing different roles and backgrounds, including decision makers, user representatives, frontline workers and mental health specialists. During the study, 31 municipalities and organizations were approached, of which 18 provided one or more contacts, and a total of 34 invitations were sent out to key informants.

Experts received per email the list of ICMs identified in the literature review and were asked to (a) select those that they were familiar with, (b) add any additional relevant models that we may have missed during the search, and (c) provide any comments about the listed and added ICMs. Responses were provided face to face, by phone or Skype video conference between March and May 2020, ranged from 20 to 111 minutes in length and lasted 44 minutes on average. The resulting list was filtered by the research team to exclude ICMs that were: (i) duplicated from literature search, (ii) of low familiarity among the key informants, (iii) missed accessible information to describe the ICMs further, or (iv) did not meet any of the three inclusion criteria used for selecting ICMs in the literature search (see above).

Two frameworks were applied to categorize the selected ICMs by degree of integration [21] and dimension of integration [6]. The concept of degrees of integration developed by Ahgren and Axelsson (2005) describes a continuum ranging from full segregation (no linkage, coordination or cooperation) to full integration (one physical colocation) [21]. The framework of the dimensions of integration by Wolfe et al. (2016) embraces vertical integration (across primary and specialist care), horizontal integration (across different sectors), longitudinal integration (across the life span) and population integration (whole system) [6].

### Identification of facilitators and barriers to successful implementation or scale up

Key informants that also participated in the previous phase were contacted and asked to participate in a semi-structured interview to assess the selected ICMs and identify facilitators and barriers to their successful implementation or scale up. Experts received the final list of ICMs with detailed descriptions before the interviews. They were then asked to comment on each of the ICMs with regards to their level of innovation [22, 23], likelihood for the innovation to be further implemented in the next 10 years [19] and the potential impact on children and youth with mental health difficulties [19, 22, 24]. These assessment criteria were included in the topic guide that was drawn up prior to the interviews together with a question about characterizing innovative ICMs; however, results of the latter are not presented herein.

Interviews were conducted by phone or Skype video conference in June 2020, ranged from 30 to 82 min in length and lasted 50 min on average. All interviews were conducted, recorded and transcribed in Norwegian by ICH. In order to present the key elements of the experts opinions, a thematic analysis [25] was conducted by ICH using NVivo. Common themes or patterns were identified by looking at regularities, convergences and divergences in data through a process of constant comparison, going back and forth between the transcripts [26]. Triangulation of analysis was conducted by the remaining two authors to enhance quality of findings.

## Results

### Sampling results

Nine experts based in six different municipalities agreed to participate in the first part of the study (identification of ICMs), and seven in the second part (assessment of selected ICMs). Six of the nine experts were based in urban areas (larger municipalities in Oslo, Viken and Vestfold og Telemark county) and three in rural areas (smaller municipalities in Innlandet and Agder county). Eight participants were female, seven had reached the age of 40 and six had gained at least 15 years of work experience in health care. Five experts worked as administrators or clinicians focusing on mental health care. One user representative, predominantly involved in mental health care, participated in the study (Table 1).

**Table 1:** Characteristics of key informants

Counties	Sex	Age	Work experience within the mental health field	Roles
• Oslo (n = 4) • Innlandet (n = 1) • Vestfold og Telemark (n = 1) • Agder (n = 2) • Viken (n = 1)	• Female (n = 8) • Male (n = 1)	• Average 45 years • Range 28–60 years	• Average 17 years • Range 3–30 years	• Administrator • Counsellor/advisor within mental health • General practitioner • Health nurse • Psychiatrist • Psychologist • User representative

**Fig. 1:**
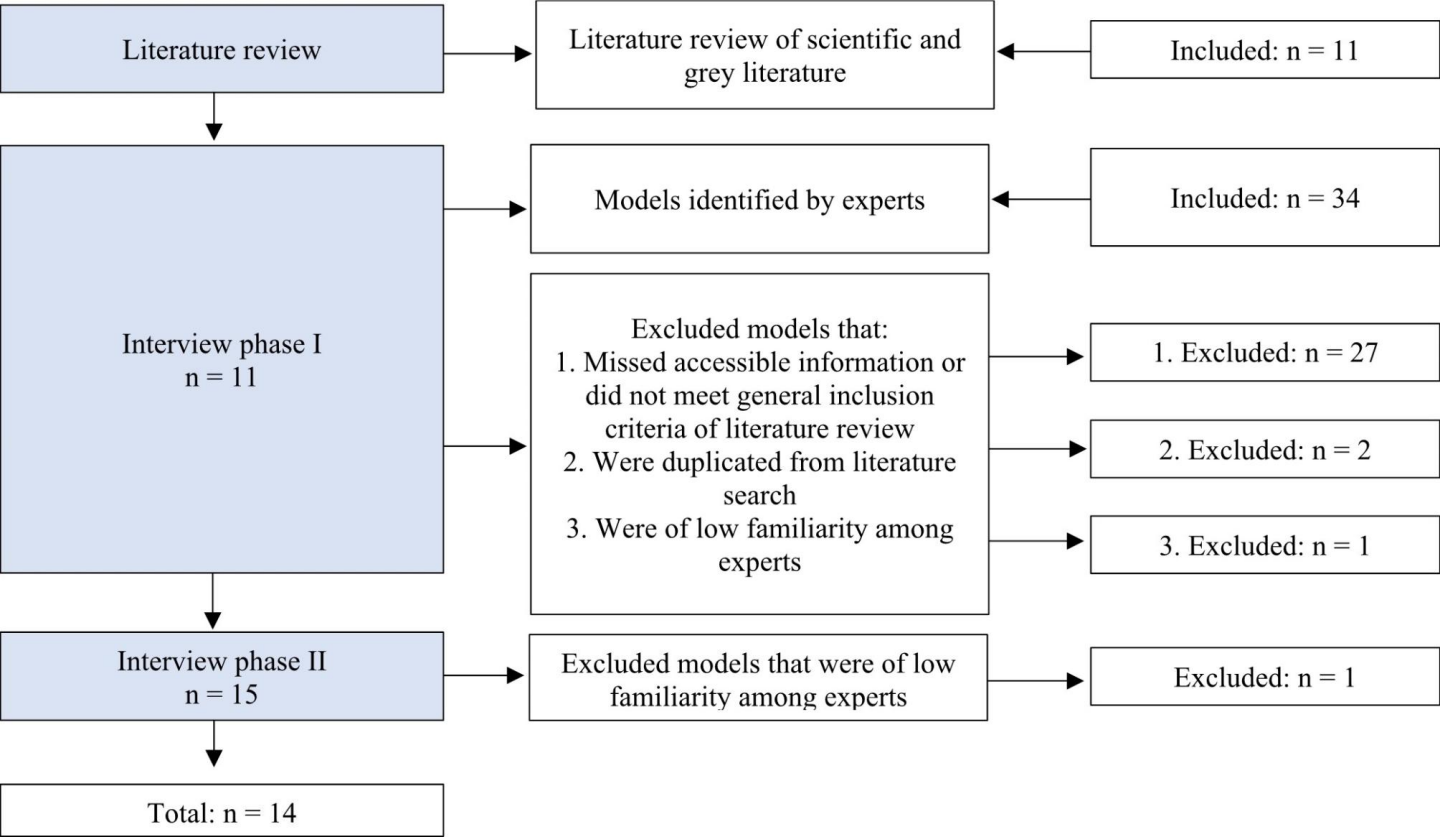
Filtering process of integrated care models

The literature search identified 11 ICMs published until February 2020. These models were presented to the experts in the first interview round. The experts identified 34 new models, of which 27 did not meet the inclusion criteria or missed accessible information and two were duplications. Furthermore, one was dropped since the experts were not familiar with it. Therefore, 15 models were selected for the second round of interviews. In the second interview round, one more model was excluded, as the informants were not familiar with it and it was not further elaborated on by the one identifying it. The filtering process resulted in 14 models being selected for further analysis (Fig. 1). Additional files present the list of total ICMs discussed (see Additional file 1 and 2).

Of the in total 14 selected ICMs, three were adapted or based on international models [30, 32, 39] (Table 2). The selected models provided different treatment philosophies ranging from self-care and community care to specialist care. Several models focused on the referral process, emphasizing low-threshold access, and the inclusion of other sectors (e.g., housing and child welfare). Four models [31, 33, 36, 38] included family or parents in their target group and five [27, 31, 32, 37, 39] involved children and youth beyond 18 years of age.

With regards to the degree of integration [21], ten models focused on *coordination in network*, i.e., they incorporated one or more coordination mechanisms between different units [28–32, 34–38]. Six models included *cooperation* – a higher degree of integration in which an appointed person aims to enhance contacts between the organisational units involved [29, 30, 32, 36, 39, 40]. Three models embodied *full integration* through physical co-location of the units [27, 33, 39]. Five ICMs also showed a mix of degrees of integration [29, 30, 32, 36, 39]. In terms of the dimensions of integration [6], all models embodied a vertical dimension; and in addition, seven models incorporated a horizontal [27, 29, 30, 32, 33, 36, 39], four a longitudinal [27, 33, 37, 39] and two a population dimension [27, 33]. Twelve models have been piloted or implemented recently (2015–2020) [27–33, 35–39]. Ten models were identified through the literature review [27, 28, 30, 32, 33, 35, 36, 38–40] and four additional models were included following experts’ suggestions [29, 31, 34, 37].

Several barriers and facilitators to successful implementation or scale up of ICMs emerged from the analysis of the expert interviews. They were related to the Norwegian healthcare system, mental health care delivery and some were more child and youth specific (Table 3).

**Table 2:** Description of selected integrated care models for child and youth mental health

Integrated care model	Start year	Objective	Description	Target group	Degree of integration*	Dimensions of integration**	Implementation status	Source
**0–26 Lier** [27]	2019	To find good solutions together with the child/youth and family, and to provide quick and the right support	• Includes family, school, health, social, economy and housing services • Offers health promotion, prevention, professional care, and self-care by an intersectoral team	Children and youth, 0–26 years and their family	Full integration	Vertical, horizontal, longitudinal and population	2019: Expansion to include children ages 0 to 12 years as well as families	Literature review
**Children and youth’s health service – Helse Fonna Health Authority** [28]	2016	To ensure children and young people receive the right help from the right service and to connect the different services to provide an integrated and comprehensive offer	• Develops seven new care pathways, ranging from concerned behaviour to specific diagnoses • Describes and maps roles and responsibility of the services offered	Children and youth with mental health challenges	Coordination in networks	Vertical	2019–2021: Implementation	Literature review
**Better mental health care for children in child welfare** [29]	2016	To develop different initiatives and models for better integrated care services for children and youth utilizing child welfare services	• Considers two models: (1) responsibility allocation, i.e., one professional being responsible for the child’s welfare and health care services, and (2) new care pathways	Children and youth in child welfare services	Cooperation (responsibility allocation) Coordination in networks (pathway)	Horizontal and vertical	(1) 2018: Started implementation of responsibility allocation and (2) 2019: Started implementation of new care pathways	Experts’ suggestion
**BTI – Better multidisciplinary efforts** [30] adapted from Denmark	2012	To create a comprehensive and coherent system between different services	• Provides a travelling log file following the child/youth • Assignes a personal coordinator	Pregnant women, children and/or youth with connected raised concern	Mix of cooperation and coordination in networks	Horizontal and vertical	Ongoing: Further developments and implementations	Literature review
**One who listens – Mental Health Youth** [31]	2016	To promote mental health and provide a place where youth can talk about their problems	• Offers free counselling by a psychologist, social worker or nurse student in cooperation with municipal services • Focuses on early intervention	Youth, 15–25 years	Coordination in networks	Vertical	Ongoing: Further developments and implementations	Experts’ suggestion
**FACT** (Flexible Assertive Community Treatment) **Young** [32] adapted from the Netherlands	2020	To ensure that young people who often “falls between two stools” receive assessment, treatment and rehabilitation services to prevent the onset of symptoms and improve quality of life, in a youth-friendly environment	• Provides multidisciplinary and flexible outreach treatment	Children and youth, 12–24 years, with complex challenges	Mix of cooperation and coordination in networks	Horizontal and vertical	2020–2023: Piloting	Literature review
**The family’s house Færder** [33]	2016	To ensure that children and youth have a good upbringing that leads to a good adult life with participation in society and work	• Includes child welfare, preventive health and school and kindergarten services	Children, youth and families	Full integration	Vertical, horizontal, longitudinal and population	Ongoing: Further developments and implementations	Literature review
**The health fellowship** [34]	2019	To ensure that hospitals and municipalities collaborate better	• Establishes arrangements between health authorities, municipalities, hospitals, GPs and patients to develop and locally adapt service models	Vulnerable patient groups, children and youth	Coordination in networks	Vertical	2020–2023: Planning and establishing	Experts’ suggestion
**Care pathway, mental health disorders – children and youth** [35]	2016	To increase user participation and satisfaction, to promote coherent and coordinated patient processes, to avoid unnecessary waiting time for assessment, treatment and follow-up, and to improve somatic health care and healthy living habits	• Coordinates GP, specialist sector, the municipality and the user or guardians • Describes contact points and follow-up care in municipalities, somatic health care and healthy living habits	Children and youth with one or more sign(s) of serious mental illness	Coordination in networks	Vertical	2018–2019: Started implementation	Literature review
**Coordination of local drugs and crime preventative measures (SLT) Bærum** [36]	2013	To prevent drug use and crime among children and youth	• Consists of a team-based collaborative model between municipality, police, community services and professionals working closely with children and youth	People at risk of committing crime and possible victims of crime – main emphasis on children, youth and their parents	Mix of coordination in networks and cooperation	Vertical and horizontal	Ongoing: Further developments and implementations	Literature review
**The Scaffolding Builders** [37]	2015	To close gaps between children and services, and arrange a comprehensive follow-up care centered around children’s needs	• Identifies gaps in the system to ultimately strengthened it	Child or youth living in foster care or residential childcare institution, 0–23 years	Coordination in network	Vertical and longitudinal	2020: Included in Akershus University Hospital’s operating framework	Experts’ suggestion
**Multi-disciplinary low-threshold team Tromsø** [38]	2015	To ensure children and young people use services that are professional, coordinated and characterized by continuity between the municipality and specialist sector	• Consists of a multidisciplinary, intersectoral outreach team • Allows initial referrals to be made by professionals as well as family members	Children, youth and families	Coordination in network	Vertical	Ongoing: Further developments and implementations	Literature review
**Youth Arena Oslo** [39] adapted from Australia	2016	To reach children and young people who are not reached by other measures, and to offer youth-friendly help and conversations	• Offers low-threshold counselling in a youth-friendly drop-in environment • Carries out multidisciplinary collaboration	Children and youth, 12–25 years	Mix of cooperation and full integration	Longitudinal, vertical and horizontal	Ongoing: Further developments and implementations	Literature review
**GP supervision groups, DPS Vestfold -** **suggested expansions to include child and adolescent psychiatric outpatient clinic (BUP)** [40]	2017	To improve collaboration in order to provide a more coherent health service, and to promote competence development so that all parties achieve the highest possible competence in patient care	• Includes services provided by the GPs, municipal psychiatry services, district psychiatric centres and the Norwegian Labour and Welfare Administration	Originally adults but suggested to be expended to children and youth	Cooperation	Vertical	2020: Report suggested the expansion of the model [40] Ongoing: Further developments and implementations	Literature review

Framework applied from *Ahgren and Axelsson (2005) [21] and **Wolfe at al. (2016) [6]

### Barriers and facilitators related to the Norwegian healthcare system

Experts highlighted two main barriers and one facilitator related to the Norwegian healthcare system. The first barrier was linked to the gatekeeping system and referral process with limited access to specialized care, which was pointed out to hinder continuity of care: *“I find it difficult to help the youth progress. They can come and talk to us, but then we don’t manage to provide them with further low-threshold psychological support” [health nurse at the municipal level].* Experts preferred ICMs that improved service flow by providing alternative solutions to the current referral process, for example, different healthcare staff were allowed to refer the user or no referrals were needed. The second barrier was related to separate legislation and financial flows between different sectors and levels of care and was considered to lead to a silo structure in the delivery of mental health care for children and youth. Experts highlighted the need to identify the silos and understand their boundaries to be able to support care coordination: *“I believe that we need quite clear silos first before we can achieve collaboration. Because when people don’t know which services they belong to or what legislation they fall under, it’s difficult to establish collaboration because we end up only discussing who should and shouldn’t do what” [administrator at the municipal level].* In addition, experts expressed the need for better vertical integration in order to enhance service flow of mental healthcare provision: *“There is likely a belief among many that it is the future – that it needs to be a much greater integration of both specialist and municipal healthcare services” [senior advisor at the national level].* A top-down approach combined with a bottom-up approach appeared to facilitate implementation or scale up of ICMs. This included the effect and need of a central push from the top:*“I actually believe that it is only when there are clear directives from the top that it will truly be broadened out, when there are specific mandates in place” [GP]*, and at the same time municipalities taking ownership of the implementation or scale up for it to be successful:*“The ownership and local anchoring are crucial in achieving the collaboration that we aim for because I believe it is easy for both us, probably also the Ministry, and others to provide guidelines on how we think this should be done. However, if no one feels they have been part of the process and cannot fully support it, we will not achieve those goals, and it will certainly take much longer” [senior advisor at the national level].*

### Facilitators related to mental health care delivery for children and youth

Experts identified four facilitators to implementation and scale up of ICMs. First, there was a general agreement that intersectoral and multidisciplinary teams facilitated implementation and coordination because they favoured the sharing of expertise and enhanced understanding of roles and responsibilities: *“(multidisciplinary low-threshold teams) have a coordinating effect because they involve different sectors and ensure interdisciplinary efforts, promote competence development, identify who can do what, and ensure patient involvement” [psychiatrist and administrator at the specialist level].* Second, providing the possibility to make adjustments to the location and time of medical appointments for children and youth seeking support enhanced availability and accessibility of mental health services: *“Ensuring that there are local places where it is easy to drop by and where one can also get assistance with difficult questions” [psychiatrist and administrator at the specialist level].*

Third, experts supported the flexible outreach model, where healthcare staff visits the user at home or in their environment, which enhanced accessibility and the delivery of tailored mental health services: *“Not everyone feels comfortable coming to our offices. There’s also something about being where people are, which gives you a different perspective on what things are about” [specialised psychologist at the municipal level].*

And forth, providing the right balance between individualized versus standardized care emerged during the interviews. Some experts showed a general underlying scepticism about mental health care delivery for children and youth being standardized because (i) individualized care delivery was considered critical for the user’s specific needs of coordinated care to be met: *“I am very aware of the negative aspect of standardizing things. It can, in a way, increase the likelihood of systematic errors” [administrator at the municipal level]*, and (ii) municipalities varied in available resources, location, and user demographics: *“I also think it’s very useful that it’s not entirely rigid and set in stone, that ‘this is how it should be’ because there are significant differences considering that municipalities vary greatly in size, extent, and other factors” [GP].* However, experts also highlighted the necessity for establishing rules and standards to avoid unwanted variation in treatment and access to care across geographical boundaries and to be less “person dependent”. Being person dependent refers to ICMs being reliant on individuals for their success: “*It is highly person-dependent to make it work. It requires a bit of a passionate and dedicated effort from the person in the position to make it function” [administrator at the municipal level].*

### Facilitators specific to children and youth

Two facilitators specific to children and youth emerged during the expert interviews. First, experts supported the development of ICMs arching over the longitudinal dimension of integration, especially in relation to the developmental stage around the age of 18, because: *“I am very fond of initiatives that extend beyond the 0–18 age range because we see that it is such a critical stage when they are transitioning into adulthood but are not yet fully adults” [senior advisor at the national level].* And second, experts considered that ICMs that embraced both the horizontal and population dimension to be a facilitator, allowing for a more holistic approach to care with an increased involvement of different sectors influencing the child or youth’s health and wellbeing (e.g., family, public health, school, housing, and work). *“Our dream here is to have some kind of house in (the municipality) where we can accommodate volunteers, and service providers from Young Arenas and BUP (Child and Adolescent Psychiatric Outpatient Clinic) can be involved. Where there are courses and various activities - a place where everything happens and there is no stigma in walking in. I believe that is the future” [specialized psychologist at the municipal level].*

**Table 3:** Findings from key informant interviews assessing integrated care models for child and youth mental health

Integrated care model	Facilitators and barriers to successful implementation or scale up	Examples of quotes
**0–26 Lier** [27]	**Facilitators** • Provides easy access (by phone, short waiting time, limited need of assessment forms to fill out)* • Provides care through intersectoral teams, creating a holistic and smooth pathway • Reaches beyond the critical age of 18** • Includes housing – especially important for children and youth not living with their parents**	*“The idea is to intervene early, with a low threshold and eliminate many assessment forms that imply that if you tick certain boxes, you will receive help.” Administrator at the municipal level **“And then, housing comes into play, which is a crucial factor and is not included in many other models because they only extend until the age of 18 and assume that children always live at home.” Senior advisor at the national level
**Children and youth’s health service – Helse Fonna Health Authority** [28]	**Facilitators** • Allocates responsibilities across different levels of care, and hence avoids unequal access to services as the service is less dependent on individuals for their success (less “person dependent”) **Barriers** • Professionals may select the wrong pathway as there are overlapping approaches • Depends on high expertise in the front line to select right pathway • Requires a certain flexibility to change pathway • May focus on a specific condition too early* • Information about the model is not directed to the users	*“How do you know which pathway to start on? Because it’s really important to jump on the right track, and for that, you need a lot of specialized knowledge and high professional competence far in advance. Otherwise, you may do injustice to children.” Administrator at the municipal level
**Better mental health care for children in child welfare** [29]	**Facilitators** • Narrows the gap between child welfare and need for mental health services • May promote cooperation between child welfare services and child and adolescent psychiatric outpatient clinic*	*“There is a significant need for it because a report stated that 9 out of 10 individuals in the child welfare system have some form of mental disorder or mental challenge, which is not surprising considering the experiences they have been through. The child welfare system says it is difficult to collaborate with the Child and Adolescent Psychiatric Outpatient Clinic (BUP), and BUP says it is challenging to collaborate with the child welfare system. There is often high pressure in the child welfare services, and there is little room for maneuver if something urgent happens.” Counsellor within mental health at the municipal level
**BTI – Better multidisciplinary efforts** [30]	**Facilitators** • Establishes basic structures for cooperation across interdisciplinary teams on a local level* • Provides systematic care delivery, avoiding unequal access to services as the service provision are to be less dependent on individuals for their success (less “person dependent”) • Is a devoted model on a national level (a central push) **Barriers** • Consider the travelling log-document a barrier for some providers to put into use • Travelling log-document may not work for complex cases • Depends on good leadership and available resources in order to be successful • Lacks innovation as the principles of this ICM is already in place locally	*“It is more of a system tool for working interdisciplinary on concerns related to children. It’s beneficial to have a common language, in a way, within the services and to have a system in place for identifying, assessing, and approaching concerns.” Health nurse at the municipal level
**One who listens – Mental Health Youth** [31]	**Facilitator** • Allows students to practise and learn while increasing accessibility to mental health services (win-win situation) • Integrates mental health services with general services in the community* **Barriers** • Students may have limited experience and expertise • Depends on the availability of students	*“I think that is a very effective way to integrate a service that may be considered taboo, such as mental health, by incorporating it into regular operations.” Senior advisor at the national level
**FACT Young** [32]	**Facilitators** • Tailors services to children and youth who need it the most, i.e. those with complex needs or those who lack a support system* • Provides flexible**, comprehensive and low threshold services (no referral needed, specialist contacting patient) • Approaches youth in their environment, providing better understanding about their context • Reaches beyond the critical age of 18 • Reconsiders competences of professionals across care levels • Supports creating a better understanding of responsibilities and collaboration across care levels • Is a devoted model on a national level (a central push) **Barrier** • Unclear funding streams in or across the different sectors involved	*“ (…) If we manage to reach more of those children and adolescents, I believe it is the most vulnerable ones who lack other support systems, whether it be family or stability.” Senior advisor at the national level **“Being able to go directly to the user instead of requiring the user to come to the services is something I strongly believe in. We need more of that.” User representative
**The family’s house Færder** [33]	**Facilitators** • Provides a good understanding of roles and responsibilities • Promotes fast collaboration as services are offered in one place* **Barriers** • Depends on anchoring and good leadership within and between different services • Requires a good understanding of the different services offered and their mandates** • Needs to implement a clear framework for collaboration	*“We can see that, especially for families, it is perceived as easier to deal with. And it is true that if you are forced to be in the same building and have offices next to each other, it becomes easier to communicate with another service, which allows for better creation of comprehensive and cohesive support for those children or families.” Senior advisor at the national level **“(…) I think such a family house requires quite clear management and understanding of the whole house so that you not only co-locate, but get the different services in the house to flourish with their mandate and make things fit together.” Administrator at the municipal level
**The health fellowship** [34]	**Facilitators** • Is implemented by large independent organizations, such as the Regional Health Authorities* • Increases competence and specialization in providing care for vulnerable patient groups • Supports merging primary with specialist care • Is a devoted model on a national level (a central push) **Barriers** • May be stigmatizing and less effective to focus on the vulnerable instead of using a broader and positive public health approach • Implemented measures may be too generic and “one size fits all” does not work • May not be innovative since this type of cooperation is considered to be already be in place**	*“What is innovative about it is that they actually require such large independent enterprises, sush at the Regional Health Authorities, to actually implement it.” Senior advisor at the national level **“It simply gives new names to things we already do. I think it may indicate that those who came up with it have a lack of knowledge about what actually happens. This includes the practice consultant scheme and the collaboration committees working between municipalities and hospitals.” GP
**Care pathway, mental health disorders – children and youth** [35]	**Facilitators** • Creates a system of the different roles and responsibilities of the providers in the care pathways • Provides clear feedback to patients and families and supports user involvement. • Increases cooperation, coordination and workflow across care levels • Elevates competencies and consciousness regarding differences between mental health difficulties and mental health disorders • Clarifies what services should be delivered by primary or specialty care • Enhances early intervention in municipalities and supports prioritisation of care for the specialist sector* • Has been well adopted by the specialist sector • Is a devoted model on a national level (a central push) **Barriers** • Provides standardized pathways that does not work for every patient • Care provision is dependent on the GP’s decisions, resulting in unequal access to services • Requires knowledge by the providers to use the right pathways** • Requires time to fill out the assessment forms by patients and families • Needs to be better anchored in the communities to avoid ad hoc solutions	*“So the care pathways reaffirm the importance of the prioritization guidelines and identifying early signs and symptoms that need to be detected for people to receive help for their optimal development. It somehow pushes us in the right direction.” Administrator at the municipal level **“While it may state in the care pathway that it should be individually tailored and comprehensive, it turns out that it doesn’t quite work that way in practice. There is a lot of coding involved, and it requires a significant number of administrative positions for registration and data entry (…).” Counsellor within mental health at the municipal level
**Coordination of local drugs and crime preventative measures (SLT) Bærum** [36]	**Facilitators** • May have a good spill over effect onto child welfare services • Supports cooperation with the police in relation to local crime preventative measures connected to mental health conditions **Barriers** • Is dependent on individuals for their success (“person dependent”) • Does not provide a clear mandate or organizational framework regarding who should have the coordinating role and carries responsibilities in the municipality* • Requires a national mandate to be further broadened out	*“It varies greatly among municipalities as well. In some, the public health coordinator also serves as the SLT (Coordination of Local Substance Abuse and Crime Prevention Measures) coordinator and does the best they can. So, apart from Bærum, we don’t have many others who are clear about how they work on this.” Administrator at the municipal level
**The Scaffolding Builders** [37]	**Facilitators** • Identifies challenges and gaps and strengthens the system* • Tailors the measures to the individual user **Barriers** • Scale up may fail due to financial strains in child welfare services.	*“It is very health-promoting and focuses on strengthening what can be strengthened. I think it is wise for someone to take responsibility for identifying where the issues lie.” Administrator at the municipal level
**Multidisciplinary low-threshold team Tromsø** [38]	**Facilitator** • Facilitates cooperation and coordination through increased understanding and shared competences between different sectors **Barriers** • May provide “too easy” access to specialized services* • Includes a small number of professionals compared to the task at hand	*“I believe it is somewhat typical for specialized healthcare services to want to work in municipalities, but are not allowed to do so. It becomes too easy to receive challenging treatment, which not everyone should have access to.” Administrator at the municipal level
**Youth Arena Oslo** [39]	**Facilitators** • Provides high accessibility (locally adapted youth-friendly environment, volunteers as part of staff) • Is a health promoting organisation bridging the gap of services between no services and specialty care* • Focuses on user participation **Barriers** • Municipalities may lack expertise and placements, and find decisional process of the organisational structure challenging • Needs to be adapted locally according to resources and expertise available	*I believe that Youth Arena is an excellent offer. It tries to fill the gap between no help and specialized healthcare services. Providing localized help where people are. I am positive to the user involvement principles and recovery thinking.” User representative
**Original ICM**: **GP supervision groups, DPS Vestfold** **Suggested expanded to include Child and adolescent psychiatric outpatient clinic** **(BUP)** [40]	**Facilitator** • Improves cooperation with the GPs and increases the effectiveness of the referral process • Includes the Norwegian Labour and Welfare Administration **Barriers** • Limited GPs’ capacity to incorporate model*	*“I believe that many who work in District Psychiatric Centers (DPS), the Norwegian Labour and Welfare Administration (NAV), Child and Adolescent Psychiatric Outpatient Clinics (BUP), and the Child Welfare Services all desire better collaboration with general practitioners (GPs). However, poor GPs have taken on so many roles that it becomes yet another role for them.” Counsellor within mental health

## Discussion

Our study selected 14 ICMs that addressed child and youth mental health in Norway, mostly by incorporating one or more coordination mechanisms between different units or by enhancing cooperation across organisations. Experts identified barriers and facilitators related to the Norwegian healthcare system, mental health care delivery, as well as services delivered specifically to children and youth. The gatekeeping system and referral process with limited access to specialized care as well as separate legislation and financial flows across organizations seem to act as a barrier to the successful implementation of the ICMs in Norway, whereas finding the right balance between a top-down and a bottom-up approach facilitates implementation by both setting clear expectations and allowing municipalities to take the lead and adapt to local conditions.

As research points out, implementation is multifaceted and a complex phenomenon, and hence does not embody universal explanations [41]. It was not within the scope of this study to go further into identifying implementation strategies for the selected ICMs, however some of the identified facilitators and barriers can be subject to further empirical implementation studies. With regards to organizational theories (organizational climate, culture and leadership), their use in empirical implementation studies has been seen as limited [41]. Also, organizational readiness for change in healthcare settings is an important factor in successful implementation of new policies, programs, and practices [42]. Therefore, further implementation studies on the selected ICMs could be subject to future research, especially within organizational theories and readiness for change.

A facilitator that emerged to be linked to the Norwegian healthcare system was the need to find a balance between flexibility and rigidity in standardization. Our study highlights the need for a rule-based system with a central top-down mandate coupled with a system trusting and giving room for local and individual adjustments and adaptations through a bottom-up approach. Providing room for individualized care while finding a balance between flexibility and rigidity in standardization are found to be crucial for the identified ICMs to emerge and adapt to the given context [43]. This balance also plays in on the aspect of counteracting unwanted variance of care and on the ICMs success. Experts highlighted the advantages of (i) allocating responsibilities across different levels of care while also (ii) providing systematic care delivery, especially when it comes to making the model less “person dependent”, which is referring to ICMs being dependent on individuals for their success.

Our results further indicate that the central government’s level of commitment to implementation of ICMs is key for their success and scale up. This can be operationalized by having a well-organized process and creating ownership at all levels. At the same time, having a centralized system or standardization presents its challenges. The responsibility of the services in the health care system is divided between the central level and the local level, and there is a tension between national ambitions and local decisions in the financing and provision of health services [44]. This tension and differences in organisation affect the local opportunities to adapt child- and youth-friendly ICMs. For example, deciding on the location and composition of personnel might vary as the municipalities have different resources and priorities to consider.

Identified facilitator related to mental health care delivery specific to children and youth focused on developmental phases and having a child- and youth-friendly approach. Our study shows ICMs adopting a child- and youth-friendly approach across the different dimensions of integration in order to meet the needs of children and youth. These findings are supported by other studies [9, 10] and trends in various countries (e.g., Australia, Ireland and Great Britain [45, 46]). Our study further underlines the importance of ICMs considering the differences between a young individual and an adult. Children and youth’s needs and approaches to care delivery differ from adults in several ways [8–10]. Relationships with parents, peers, school, work, leisure and other key persons and arenas in their life evolve and change as they get older and move through various developmental stages. Their developmental phases and age also result in different legal status, e.g., before and after legally entering adulthood, and affect what kind of services they might receive. Our study shows five different models aiming to coordinate and support the youth in the transition from “child” to “adult” services by specifically arching over the critical age of 18 years [27, 31, 32, 37, 39]. Several of the identified ICMs handle the shown complexity by offering a child- and youth-friendly approach through (a) providing accessible services and (b) being holistic. With regards to accessibility, this study points to different measures, e.g., access by phone, short waiting time and low-threshold meetings, and for the ICMs to be holistic, they need to take into account the different dimensions of integration [10].

Two study limitations warrant consideration. First, several models were not assessed by the experts because of lack of familiarity or lack of accessible information. Given that the study objective was to identify and assess emerging ICMs, which may not have gained popularity yet, this limitation was expected and counted for in the study [18]. And second, the participation rate was only 26% (nine experts out of the 34 invited agreed to participate) and therefore the results may not represent the full spectrum of opinions about the selected ICMs, i.e. data saturation may not have been reached. The low participation rate is probably due to the fact that the study took place during a national lockdown as a response to the COVID-19 pandemic. Furthermore, participants most likely struggled to keep up with the increased need for mental health services and had less time available to participate in research. We applied a snowball technique to reach more participants, however, we were not able to recruit more than nine experts within the limited timeframe of the study. Nevertheless, a heterogeneous sample of informants participated in the study and interviews elicited interesting and relevant results that helped to identify and assess promising ICMs for children and youth, including barriers and facilitators to implementation and scale up.

Our study shows two main strengths. First, the chosen method obtained the right information through open reflections and diversity in opinions gained from a heterogeneous sample of key informants. The interviews provided in-depth knowledge on how ICMs can be innovative and what factors can favour or hinder implementation or scale up of ICMs for children and youth. Second, new evidence shows that the COVID-19 pandemic led to a global rise in depressive and anxiety disorders in 2020, with the highest change in prevalence among the younger age groups, making the study topic even more relevant [47].

## Conclusion

Care delivery targeting the needs of children and youth with mental health difficulties requires further adaptation to accommodate the intricate nature of their lives. They are not “small” adults, and different needs require different solutions. ICMs have been identified as a means to address this complexity by offering accessible services and adopting a holistic approach. In addition to the importance of having a child- and youth-friendly approach, experts provided in-depth knowledge on the balance of individualized versus standardized care delivery, the tension between national ambitions and local decisions and different aspects of system fragmentation. This study highlights a selection of promising ICMs that appear capable of meeting some of the specific needs of children and youth. However, it is recommended to subject these models to future research and evaluation for further assessment and refinement to ensure their effectiveness and the fulfilment of their intended outcomes. It could also be interesting for future research to conduct a cross national comparison on barriers and facilitator specific to children and youth with similar systems as in Norway.

### Electronic supplementary material

Below is the link to the electronic supplementary material.

Supplementary Material 1

Supplementary Material 2

## Data Availability

The datasets supporting the conclusions of this article are included within the article and its additional files.
