# Supplementary material for: Emerging integrated care models for children and youth with mental health difficulties in Norway: a horizon scanning study
Source: BMC Health Serv Res. 2023 Aug 14;23:860. doi: 10.1186/s12913-023-09858-x (PMC10426212; doi:10.1186/s12913-023-09858-x)
Supplement: Supplementary file 1 — Supplementary Material 1 [file 12913_2023_9858_MOESM1_ESM.docx]

Additional file 1

List of Integrated Care Models (ICMs) for children and youth

*Identified in the scientific and grey literature review:*

| **Title (English translation)^[[1]](#footnote-1)^** | **Location** |
| --- | --- |
| Care pathway, mental health disorders – children and youth | Norway |
| Coordination of local drugs and crime preventative measures | Bærum, Norway |
| Better multidisciplinary efforts | Norway |
| The family’s house | Færder, Norway |
| Children and youth’s health service – Helse Fonna Health Authority | Sunnhordland, Indre Hardanger and North Rogaland, Norway |
| FACT (Flexible Assertive Community Treatment) Young | Norway |
| Youth Arena | Oslo, Norway |
| 0-26 Lier | Lier, Norway |
| Care pathway: when concerning absence from school becomes a severeloss of function | Trondheim, Norway |
| Multidisciplinary low-threshold team (partly ambulatory) | Tromsø, Norway |
| Expansion of existing GP supervision groups to include cases from child and adolescent psychiatric outpatient clinic | Sandefjord and Larvik, Norway |

*Models identified by experts:*

| **English (translation)^1^** | **Location** |
| --- | --- |
| Better mental health care for children in child welfare | Norway |
| The health fellowship | Norway |
| The Scaffolding Builders | Norway |
| Health-promoting kindergartens and schools | Agder, Norway |
| Increased collaboration between hospital/medical center and child and adolescent psychiatric outpatient clinic/psychologist competence | Norway |
| Professional center for children and young people in Alna District | Alna, Norway |
| One who listens (Mental Health Youth) | Norway |
| Multidisciplinary low-threshold team | Norway |
| The help of Stange | Stange, Norway |
| “Expanded” Youth Arena | Norway |
| The Norwegian Directorate of Health user council within mental health and substance abuse | Norway |
| A Norwegian health nurse (Tale M. K. Engvik) on social media | Norway |
| Interdisciplinary statement of consent | Norway |
| E-mastering tool | Norway |
| S.H.A.R.E. (Support local organizations, Hire in mentorship programs, Ambassadors, Raise awareness and Empowerment) | Norway |
| The new Asker | Asker, Norway |
| Mental health of young farmers | Norway |
| Dialogue as part of the referral process | Norway |
| Health station for gender and sexuality | Oslo, Norway |
| Young jobseeker | Norway |
| Olafiaklinikken | Oslo, Norway |
| The RV (Mental Health Youth) | Norway |
| Modum Bad | Norway |
| The health professionals | Norway |
| Circle of security (COS) | Norway |
| ICDP (International Child Development Program) | Norway |
| Universal programs in school | Norway |
| Coping children | Norway |
| Health care project for children in child welfare institutions | Norway |
| The cooperation body | Kristiansand, Norway |
| Care and responsibility for somatic health and living habits in the care pathway for mental health and substance abuse | Norway |
| The energy center for children and young people | Bergen, Norway |
| Children in families with substance abuse | Norway |
| Love and boundarier | Norway |

1. Original titles (in Norwegian) is provides in Additional file 2. [↑](#footnote-ref-1)
