# Supplementary material for: Emerging integrated care models for children and youth with mental health difficulties in Norway: a horizon scanning study
Source: BMC Health Serv Res. 2023 Aug 14;23:860. doi: 10.1186/s12913-023-09858-x (PMC10426212; doi:10.1186/s12913-023-09858-x)
Supplement: Supplementary file 2 — Supplementary Material 2 [file 12913_2023_9858_MOESM2_ESM.docx]

Additional file 2

List of Integrated Care Models (ICMs) for children and youth (original titles in Norwegian)

*Identified in the scientific and grey literature review:*

| **Original title (Norwegian)** |
| --- |
| Pakkeforløp, Psykiske lidelser – barn og unge |
| Samordning av lokale rus og kriminalitets- forebyggende tiltak (SLT-modellen), Bærum |
| Bedre Tverrfaglig Innsats (BTI) |
| Familiens hus, Færder |
| Barn og unges helseteneste, Helse Fonna HF |
| FACT Ung |
| Ung Arena, Oslo |
| 0-26 Lier |
| Pasientforløp: "Når bekymringsfullt skolefravær blir alvorlig funksjonstap", Trondheim |
| Tverrfaglig lavterskelteam (delvis ambulerende), Tromsø |
| Utvidelse av eksisterende veiledningsgrupper for fastleger til å gjelde saker fra barne- og ungdomspsykiatrien (BUP), Sandefjord og Larvik |

*Models identified by experts:*

| **Original title (Norwegian)** |
| --- |
| "Bedre helsehjelp til barn i barnevernet" |
| Helsefellesskapene |
| Stillasbyggerne |
| HBS Agder (Helsefremmede barnehager og skoler) |
| Økt samarbeid mellom sykehus/legesenter og BUP/psykologkompetanse |
| Fagsenter for barn og unge i bydel Alna |
| "En som lytter" (Mental Helse Ungdom) |
| Tverrfaglig lavterskelteam |
| Stangehjelpa |
| "Utvidet" Ung Arena |
| BrukerROP |
| “Helsesista” |
| Tverrfaglig samtykkeerklæring |
| E-mestringsverktøy |
| S.H.A.R.E. |
| Nye Asker |
| Unge bønders psykiske helse |
| **"**Dialog som ledd i henvisningsprosessen" (Helsedirektoratet) |
| Helsestasjon for kjønn og seksualitet, Oslo |
| Ung arbeidssøker (Forandringshuset) |
| Olafiaklinikken |
| Bobilen (Mental Helse Ungdom) |
| Modum Bad |
| Helseproffer |
| Circle of security (COS) |
| ICDP (International Child Development Program) |
| Universelle programmer i skolen |
| Mestrende Barn |
| **"**Prosjekt helsehjelp til barn i barneverninstitusjoner" |
| Samarbeidsorganet, Kristiansand |
| Ivaretakelse og ansvar for oppfølging av somatisk helse og levevaner i pakkeforløp for psykisk helse og rus |
| Energisenteret for barn og unge, Bergen |
| BIR (Barn i rusfamilier) |
| Kjærlighet og grenser (KoRUS) |
